# Health Behaviors and Associated Sociodemographic Factors in Cervical Cancer Survivors Compared with Matched Non-Cancer Controls

**DOI:** 10.1371/journal.pone.0160682

**Published:** 2016-08-16

**Authors:** Boyoung Park, Se Ik Kim, Sang-Soo Seo, Sokbom Kang, Sang-Yoon Park, Myong Cheol Lim

**Affiliations:** 1 Department of Cancer Control and Policy, Graduate School of Cancer Science and Policy, National Cancer Center, Goyang-si, Korea; 2 Cancer Early Detection Branch, National Cancer Control Institute, National Cancer Center, Goyang-si, Korea; 3 Department of Obstetrics and Gynecology, Seoul National University College of Medicine, Seoul, Korea; 4 Center for Uterine Cancer, Hospital, National Cancer Center, Goyang-si, Korea; 5 Gynecologic Cancer Branch, Research Institute, National Cancer Center, Goyang-si, Korea; Baylor College of Medicine, UNITED STATES

## Abstract

We explored the prevalence of smoking, alcohol consumption, physical activity, and obesity in cervical cancer survivors and examined associations between sociodemographic factors and each health behavior. We studied 448 cervical cancer survivors ≥2 years after their initial diagnosis who had completed treatment. The total sample consisted of these survivors, and 4,480 cancer-free controls who were grouped into 5-year age cohorts and matched to the survivors in terms of both education and monthly household income. The prevalence of current smoking, current alcohol consumption, physical inactivity, and obesity in cervical cancer survivors (2.68, 23.88, 62.02, and 32.81%, respectively) did not differ significantly from those of matched non-cancer controls. Age (younger), marital status (married), and education (≥college) were associated with lower probabilities of current alcohol consumption (odds ratio [OR] = 0.91, 95% confidence interval [CI] = 0.88–0.95; OR = 0.42, 95% CI = 0.23–0.78; OR = 0.49, 95% CI = 0.25–0.97, respectively). A monthly household income ≥$2,000, being employed, and self–rated health status (less healthy) were associated with physical inactivity (OR = 0.61, 95% CI = 0.37–0.99; OR = 2.16, 95% CI = 1.36–3.42; OR = 1.94, 95% CI = 1.23–3.05, respectively). Both age and number of years since diagnosis were associated with obesity (OR = 1.04, 95% CI = 1.01–1.08; OR = 0.38; 95% CI = 0.20–0.72, respectively). The health behaviors of cervical cancer survivors did not differ from those of matched cancer-free controls. As health behaviors are modifiable, identification of cervical cancer survivors who are at risk of an unhealthy lifestyle would allow individual- and population-based intervention programs to more effectively use their limited resources.

## Introduction

Cervical cancer is the fourth most common cancer as well as the fourth most common cause of female cancer deaths worldwide. Cancer incidence and mortality rates vary geographically, and most cancers and associated deaths occur in less-developed regions [1]. Although the incidence of, and mortality from, cervical cancer have declined since the development of widespread screening programs, improved treatment, and most recently, the introduction of a vaccine against human papillomavirus, the disease burden remains high, and the numbers of long-term survivors are increasing [2]. In Korea, the incidence of cervical cancer has declined rapidly, and more cervical cancers are detected early because of the introduction of cervical cancer screening programs [3, 4]. However, cervical cancer remains the fifth most common cancer in the country and is associated with the largest number of long-term survivors [5].

Cancer survivors are at increased risk of sequelae of the cancer per se as well as of the cancer treatment: secondary cancer caused by radiotherapy, early ovarian failure attributable to surgery and/or radiotherapy, and non-cancer-related problems, including functional limitations, poor general health, and a reduced quality of life [6–9]. Cervical cancer survivors with known risk factors, such as human papillomavirus infection and smoking, are at a greater risk of secondary malignancies than are other cervical cancer survivors [10]. Healthful behaviors, such as smoking cessation, increased physical activity, consumption of a healthy diet, and weight control, can play roles in improving the long-term health-related outcomes of cancer survivors [11–13]. In addition, positive associations between the extent of physical activity and quality of life and between physical activity and other healthy behaviors have been found among cervical cancer survivors [14].

Although several population-based studies have evaluated the health behaviors of cancer survivors [6, 15–17], only a few focused on the behaviors of cervical cancer survivors in particular. As cancer survivors differ in terms of disease characteristics, risk factors, and health behaviors exhibited after diagnosis and treatment, a better understanding of the health behaviors and associated sociodemographic factors of cervical cancer survivors would allow physicians and public health professionals to provide effective individual- and population-based interventions to improve their health status and quality of life. Therefore, we used a population-based dataset to investigate the prevalence of smoking, alcohol consumption, physical activity, and obesity in cervical cancer survivors and to examine associations between sociodemographic factors and each health behavior.

## Materials and Methods

### Data source and study population

We used baseline research data from the Health Examinee (HEXA) cohort, a component of the Korea Genome Epidemiology Study (KoGES). KoGES is an ongoing population-based cohort study that commenced in 2001 and explores the effects of various risk factors and their interactions on the development of major chronic diseases in Koreans. The health examination centers in 14 urban areas of Korea collaborated (as the HEXA cohort) to recruit health examinees from 2004 to 2013. We used their baseline data. All participants provided their written informed consent. After informed consent was obtained, each participant completed a questionnaire given to him or her by an interviewer. The Institutional Review Board of the National Cancer Center approved the study protocol (approval no. NCC2014-0098). The questionnaire explored medical history and sociodemographic and behavioral characteristics and included a validated food-frequency questionnaire. In addition, each participant underwent a comprehensive physical examination, including anthropometry and biochemical evaluation of a fasting blood sample as a routine health examination. The KoGES and the HEXA cohort have been described elsewhere [18]; details may also be found on the Korea National Institute of Health website (http://www.nih.go.kr/NIH/eng/main.jsp).

Of all female participants, 618 reported (when responding to questions on medical history) that they had been diagnosed with cervical cancer by a physician. Among such subjects, survivors were defined as those who reported that their treatment had ended ≥2 years from the time of initial diagnosis as in a previous study [19]; 448 subjects were included in analysis. The comparison group was randomly selected, and it included females with no history of any type of cancer; this group was frequency-matched at a 1:10 ratio with the cervical cancer survivors and categorized according 5-year age cohort, educational level (less than high school, high school, or college or higher), and monthly household income (less than or more than $2,000 per month). Thus, we included 448 cervical cancer survivors and 4,480 non-cancer controls in our final analysis.

### Variables

Health behaviors, including smoking, alcohol consumption, and physical activity, were assessed using a structured questionnaire. Subjects who reported that they had smoked ≥ 100 cigarettes in their lifetime and who currently smoked were defined as current smokers. For drinking, the questions used were “Have you ever drunk an alcoholic beverage?” and “Do you currently drink?” For those who answered that they had drunk alcohol, the duration, frequency, and amount were additionally asked. Participants who reported that they currently consumed alcohol were defined as current drinkers. For physical exercise, participants were asked about the frequency of exercise per week and duration in minutes per exercise bout. We multiplied the frequency of exercise per week by the duration to estimate the total amount of exercise per week. Those who reported that they performed activities that caused them to sweat for ≥150 min per week were defined as physically active based on current United States [20], United Kingdom [21], and World Health Organization guidelines [22]. Body mass index (BMI), which reflects obesity status, was calculated from the anthropometric measurements; subjects with a BMI ≥25 kg/m^2^ were considered to be obese as the criterion for Asians, which is lower than that for Western countries (BMI ≥30 kg/m^2^) [23].

Other covariates included marital status (married, cohabitating, divorced, widowed, unmarried), employment status (currently employed, currently unemployed), self-rated health status (healthy, normal, unhealthy), family history of any cancer (no, yes), and other physician-diagnosed chronic disease, including hypertension, diabetes, dyslipidemia, stroke, transient ischemic attack, angina, myocardial infarction, colon polyps, fatty liver, chronic liver disease, liver cirrhosis, gallbladder stones, cholecystitis, thyroid disease, arthritis, or osteoporosis. Those who reported having any of these chronic diseases were classified as having a comorbid condition.

### Statistical analysis

The sociodemographic characteristics and health behaviors (smoking, alcohol consumption, physical inactivity, and obesity) of cervical cancer survivors and matched non-cancer controls were compared using chi-squared tests or t-tests. To explore whether cervical cancer survivors had better or worse health outcomes than the matched non-cancer controls, conditional logistic regression was performed [24] after matching for age, education, and monthly household income and adjusting for other covariates including marital status, employment status, self-rated health status, family history of cancer, presence of a comorbid chronic disease, and other outcomes (smoking, drinking, physical exercise, and obesity).

We used multiple logistic regression to assess the effects of various sociodemographic and other health-related variables on the risk of alcohol consumption, physical inactivity, and obesity among survivors. The variables included in the multiple logistic regression were age, marital status, level of education, monthly household income, employment status, self-rated health status, family history of cancer, presence of a comorbid chronic disease, years since diagnosis of cervical cancer, smoking, and other outcomes (drinking, physical exercise, and obesity). We calculated odds ratios (ORs) using these variables. All statistical analyses were performed with SAS software version 9.1 (SAS Institute, Cary, NC, USA).

## Results

The sociodemographic characteristics of the 448 cervical cancer survivors and the 4,480 matched non-cancer controls are shown in Table 1. The cervical cancer survivors did not differ from the matched non-cancer controls with regard to age, educational attainment, and household income. The proportions of divorced, widowed, and unmarried subjects were higher (P = 0.030) in cervical cancer survivors compared with non-cancer controls. The groups did not significantly differ in terms of other sociodemographic characteristics.

**Table 1:** Comparison of basic characteristics between cervical cancer survivors and female respondents never diagnosed as having cancer.

Characteristics ^a^	Survivors	Non-cancer controls	P-value ^b^
	(N = 448)	(N = 4,480)	
Age, mean (95% CI)	56.1 (55.4–56.8)	56.0 (55.8–56.3)	0.803
Age, N (%)			
<50	92 (20.5)	920 (20.5)	1.000
50–54	99 (22.1)	990 (20.1)	
55–59	92 (20.5)	920 (20.5)	
≥ 60	165 (36.8)	1650 (36.8)	
Marital status, N (%)			
Married, cohabitation	354 (79.0)	3700 (82.6)	0.032
Divorced, widow, unmarried	94 (21.0)	756 (16.9)	
Missing	0 (0.0)	24 (0.5)	
Educational attainment, N (%)			
≤ High school	370 (82.6)	3684 (82.2)	0.858
≥ College	76 (17.0)	739 (16.5)	
Missing	2 (0.5)	57 (1.3)	
Monthly household income, N (%)			
< 2000$	180 (40.1)	1800 (41.3)	0.986
≥ 2000$	205 (45.8)	2054 (47.1)	
Missing	63 (14.1)	509 (11.7)	
Employment state, N (%)			
Currently unemployed	292 (65.2)	2853 (63.7)	0.909
Currently employed	152 (33.9)	1503 (33.5)	
Missing	4 (0.9)	124 (2.8)	
Self-rated health status, N (%)			
Healthy	130 (29.0)	1488 (33.2)	0.074
Normal-unhealthy	313 (69.9)	2949 (65.8)	
Missing	5 (1.1)	43 (1.0)	
Co-morbid chronic disease ^a^, N (%)			
Yes	203 (45.3)	2248 (50.2)	0.050
No	245 (54.7)	2232 (49.8)	
Family history of cancer (any), N (%)			
Yes	143 (31.9)	1241 (27.7)	0.056
No	303 (67.6)	3225 (72.0)	
Missing	2 (0.5)	14 (0.3)	
Age at cervical cancer diagnosis, N (%)			
< 40	117 (26.1)		
40–49	222 (49.6)		
≥ 50	109 (24.3)		
Years since diagnosis, N (%)			
2–5	88 (19.6)		
6–10	136 (30.4)		
≥ 11	224 (50.0)		

CI: confidence intervals

^a^ Chronic disease includes any of following diseases diagnosed by a physician; hypertension, diabetes, dyslipidemia, stroke, transient ischemic attacks, angina or myocardiac infarction, colon polyp, fatty liver, chronic liver disease or liver cirrhosis, gallbladder stone or cholecystitis, thyroid disease, arthritis, and osteoporosis

^b^ Chi-square test or t-test results excluding the missing data

The prevalences of smoking, alcohol consumption, physical inactivity, and obesity were 2.7%, 23.9%, 62.0%, and 61.1%, respectively, in survivors, and these figures did not differ significantly from the comparable ones in matched non-cancer controls (2.2%. 26.4%, 64.6%, and 58.6%, respectively; Table 2). After adjusting for the matching variables and other covariates, the conditional logistical ORs for cervical cancer survivors compared with matched non-cancer controls were 1.11 (95% confidence interval [CI] = 0.60–2.06) in terms of smoking, 1.12 (95% CI = 0.94–1.52) in terms of alcohol consumption, 0.85 (95% CI = 0.69–1.05) in terms of physical inactivity, and 1.10 (95% CI = 0.89–1.35) in terms of obesity. Thus, the health behaviors of the two groups did not differ significantly.

The duration since diagnosis did not affect the prevalence of smoking, alcohol consumption, or physical inactivity. However, obesity decreased significantly in the 6–10 years after diagnosis, but it increased after 11 years (P = 0.004, not shown).

**Table 2:** Health behaviors in cervical cancer survivors compared with female respondents never diagnosed as having cancer.

Health behaviors ^a^	Survivors	Non-cancer controls	Odds ratio ^b^
	N (%)	N (%)	(95% confidence intervals)
Current smoking			
No	436 (97.3)	4359 (97.8)	1
Yes	12 (2.7)	99 (2.2)	1.11 (0.60–2.06)
Current drinking			
No	341 (76.1)	3277 (73.6)	1
Yes	107 (23.9)	1175 (26.4)	1.12 (0.94–1.52)
Physical activity			
Active ^c^	169 (38.0)	1523 (35.4)	1
Inactive	276 (62.0)	2775 (64.6)	0.85 (0.69–1.05)
Body mass index			
< 25Kg/m^2^	173 (38.9)	1850 (41.4)	1
≥ 25Kg/m^2^ (Obese)	272 (61.1)	2616 (58.6)	1.10 (0.89–1.35)

^a^ For smoking 22 missing in controls; for drinking 28 missing in controls; for physical activity, 3 missing in cases and 182 missing in controls; for body mass index, 3 missing in cases and 14 missing on controls

^b^ Odds ratio from conditional logistic regression after adjusting for including marital status, employment status, self-rated health status, family history of cancer, presence of another comorbid chronic disease, and other outcomes (smoking, drinking, physical exercise, and obesity)

^c^ Those who reported performing ≥150 min of sweat-inducing exercise per week

Table 3 shows the associations of sociodemographic characteristics with other health-related factors and each health outcome in survivors. As current smokers were few in number (2.68%; 12/338), we excluded current smoking when performing multivariate logistic regression analysis. Current alcohol consumption decreased age (OR for 1-year age increment = 0.91, 95% CI = 0.88–0.95), with status as married or cohabitating (OR = 0.42, 95% CI = 0.23–0.78), and in highly educated survivors (OR = 0.49, 95% CI = 0.25–0.97). Physical inactivity was lower in survivors with a monthly household income ≥$2000 (OR = 0.61, 95% CI = 0.37–0.99), but higher in those who were currently employed and those with poor subjective health (OR = 2.16, 95% CI = 1.36–3.42; OR = 1.94, 95% CI = 1.23–3.05, respectively). Obesity increased with age (OR for 1-year age increment = 1.04, 95% CI = 1.01–1.08) and was less prevalent in cancer survivors diagnosed 6–10 years prior to the study, who were less likely to be obese (OR = 0.38, 95% CI = 0.20–0.72) compared with survivors diagnosed 5 years or less prior to the study.

**Table 3:** Adjusted odds Ratio of health behaviors for each sociodemographic characteristic among the survivors of cervical cancer.

Characteristics	Current drinking		Physical inactivity ^a^		Obesity ^b^	
	Odds ratio (95% confidence intervals)	P-value	Odds ratio (95% confidence intervals)	P-value	Odds ratio (95% confidence intervals)	P-value
Age, 1 year increment	0.91 (0.88–0.95)	<0.001	1.01 (0.98–1.05)	0.428	1.04 (1.01–1.08)	0.026
Marital status						
Divorced, widow, unmarried	1		1		1	
Married, cohabitation	0.42 (0.23–0.78)	0.005	0.65 (0.38–1.14)	0.133	1.37 (0.79–2.36)	0.261
Educational attainment						
≤ High school	1		1		1	
≥ College	0.49 (0.25–0.97)	0.042	1.20 (0.68–2.13)	0.524	0.78 (0.42–1.44)	0.430
Monthly household income						
< 2000$	1		1		1	
≥ 2000$	1.58 (0.89–2.80)	0.120	0.61 (0.37–0.99)	0.048	0.98 (0.59–1.62)	0.924
Employment state						
Currently unemployed	1		1		1	
Currently employed	0.97 (0.58–1.61)	0.891	2.16 (1.36–3.42)	0.001	1.07 (0.67–1.71)	0.771
Self-rated health status						
Healthy	1		1		1	
Normal-unhealthy	0.63 (0.38–1.06)	0.079	1.94 (1.23–3.05)	0.004	0.80 (0.50–1.27)	0.335
Family history of cancer (any type)						
No	1		1		1	
Yes	1.07 (0.65–1.78)	0.780	1.00 (0.64–1.55)	0.995	0.85 (0.54–1.34)	0.473
Co-morbid chronic disease ^c^						
No	1		1		1	
Yes	1.41 (0.84–2.37)	0.191	0.78 (0.50–1.21)	0.259	1.26 (0.80–1.98)	0.323
Years since diagnosis						
≤ 5	1		1		1	
6–10	0.95 (0.48–1.86)	0.880	1.15 (0.63–2.08)	0.655	0.38 (0.20–0.72)	0.003
≥ 11	1.18 (0.62–2.27)	0.611	1.36 (0.76–2.41)	0.301	0.75 (0.42–1.32)	0.316
Current smoking						
No	1		1		1	
Yes	2.20 (0.64–7.59)	0.211	1.95 (0.50–7.66)	0.341	1.11 (0.31–4.06)	0.872
Current drinking						
No			1		1	
Yes			0.85 (0.52–1.40)	0.526	0.91 (0.54–1.53)	0.729
Physical activity						
Inactive	1				1	
Active	1.18 (0.72–1.93)	0.506			0.70 (0.45–1.10)	0.125
Body mass index						
< 25Kg/m^2^	1		1			
≥ 25Kg/m^2^	0.93 (0.55–1.56)	0.781	1.42 (0.91–2.22)	0.127		

^a^ Those who reported performing <150 min of sweat-inducing exercise per week

^b^ Body mass index ≥ 25Kg/m^2^

^c^ Chronic disease includes any of following diseases diagnosed by a physician; hypertension, diabetes, dyslipidemia, stroke, transient ischemic attacks, angina or myocardiac infarction, colon polyp, fatty liver, chronic liver disease or liver cirrhosis, gallbladder stone or cholecystitis, thyroid disease, arthritis, and osteoporosis

## Discussion

As cervical cancer survival rate has greatly improved and the number of long-term survivors has increased, problems with health behaviors and their long-term effects and management in cervical cancer survivors has been raised as important issue. In this study, we identified that cervical cancer survivors and matched non-cancer controls did not differ significantly with regard to smoking status, alcohol consumption, physical inactivity, or obesity.

The factors influencing unhealthy behaviors differed by behavior. Alcohol drinking was negatively correlated with educational level, which is consistent with previous data on cancer survivors who had undergone hematopoietic cell transplantation [25]. Physical inactivity was also inversely correlated with income, as noted previously [17]. In addition, married or cohabiting survivors drank less, and those who were employed or considered themselves unhealthy were more likely to be physically inactive, suggesting that survivors with lower education, lower income, unhealty subjective health status, being unmarried/uncohabitated, or employed were particularly vulnerable to the effects of unhealthy lifestyles. However, no sociodemographic factor studied was associated with obesity. Although previous studies identified several unhealthy behaviors were correlated in general populations [26] and in colon cancer [27] and gynecological cancer survivors [14], no interrelationship among unhealthy behaviors was observed in cervical cancer survivors in the current study, confirming modification of each health behaviors needs to be accessed individually.

An earlier study found that survivors of gynecological cancer had a higher current smoking rate than did non-cancer controls, but neither alcohol consumption nor level of physical activity differed between the two groups [28]. Another study found no significant difference with respect to smoking, drinking, or physical activity between all cancer survivors and non-cancer controls but reported that cervical cancer survivors generally exhibited more unhealthy behaviors [17]. Thus, cervical cancer survivors might be expected to exhibit more unhealthy behaviors than other cancer survivors.

Several previous studies reported that the smoking prevalence among cervical cancer survivors was higher than that among survivors of other types of cancers or in general populations [14–16]. According to research conducted in Western countries, survivors of cervical or gynecological cancer exhibited higher smoking rates (21.5–46.0% in the USA [15, 16, 29]; 20.9% in Australia [14]) than did our study population (2.7%). In Korea, the female smoking rate is the lowest (4.3%) of all countries in the Organization for Economic Cooperation and Development [30]. Additionally, our study subjects were voluntary health examinees and thus were health-conscious, which explains the lower smoking rates compared with those in other studies of both the cervical cancer survivors and the matched non-cancer controls. One previous study reported that 56.9% of cancer survivors consumed alcohol regularly [15]. This figure is higher than that in the present study, which is more consistent with the finding reported by another study: that the prevalence of risky drinking in cervical cancer survivors was only 7.2% [16]. The proportions of cervical cancer survivors engaging in physical activity have varied among studies as have the definitions of such activity. About 50% of survivors engaged in the recommended level of physical activity [19]; this was lower than the proportion noted in the general population. In other studies, 20–30% of survivors were physically active [15, 16]. Our figure is intermediate, as it is similar to that of Beesely et al. [14]. The prevalence of obesity and overweight status in Korea noted in the present and previous studies are comparable, at 20–40% [14, 29].

A cancer diagnosis may motivate an individual to adopt healthy behaviors [31]; a higher prevalence of positive behaviors was evident in cancer survivors than in cancer-free subjects in several earlier studies [17, 32, 33]. However, consistent with other studies [17, 28], we found that such behaviors were not evident in cervical cancer survivors. Thus, the “teachable moment” used to trigger healthy behavior in cancer survivors may be difficult to identify in cervical cancer survivors.

To the best of our knowledge, this population-based study is the first to examine the health behaviors, associated sociodemographic factors, and other health-related issues in cervical cancer survivors, a population that is vulnerable to effects of unhealthy behaviors.

Although we have gathered valuable information, several limitations should also be noted. First, our cross-sectional approach using a baseline questionnaire of cohort study can measure the prevalence of various health behaviors and differences between groups, but it is difficult to infer causality. Second, all cervical cancer survivors and matched non-cancer controls had voluntarily visited health examination centers for health check-ups. Therefore, they may be more concerned about their health status and practice better health behaviors than cancer survivors in general or general populations. For example, one previous study found that cervical cancer survivors recruited from tertiary hospitals had more comorbid chronic diseases than did general female populations [34]. However, in the present study, the proportion of cervical cancer survivors with comorbidities was not significantly different from controls. Thus, our results may not be readily generalizable. However, patients who complete treatment and undergo medical check-ups in health examination centers rather than cancer treatment hospitals may more readily return to daily life and thus may be prime targets for community-level efforts to provide management for cervical cancer survivors. To ensure the comparability of health behaviors, we matched survivors and controls by age, educational level, and income. We defined cervical cancer survivors using a self-report method. Although some information bias may have been affected our results, a previous study found that a self-reported cancer history was reliable [35].

In conclusion, we found that the health behaviors of cervical cancer survivors were no worse than those of matched non-cancer controls. The findings of a previous report [17] suggest that the health behaviors of cervical cancer survivors may, in fact, be worse than those of other cancer survivors. Several sociodemographic characteristics were associated with unhealthy behaviors among cervical cancer survivors, but we found no interrelationship among such behaviors. Thus, those at high risk for each unhealthy behavior differed, and interventions should be designed to modify lifestyles and interventions targeting each unhealthy behavioral factor of survivors with particular sociodemographic characteristics may be more appropriate for cervical cancer survivors. Efforts to induce healthy behavior in cervical cancer survivors are unfortunately not routine during cancer treatment and rehabilitation. Yet, health behaviors are modifiable. The identification of cervical cancer survivors at higher risk of an unhealthy lifestyle who would thus benefit from interventions would allow individual- and population-based programs to use their limited resources more effectively.
